# Knowledge-Guided Deep Learning with Clinical EEG Biomarkers for Automated Dementia Detection and Staging

**DOI:** 10.3390/diagnostics16182912

**Published:** 2026-09-09

**Authors:** Nebras Sobahi, Salih Taha Alperen Özçelik, Abdulkadir Şengür, Hanifi Güldemir

**Affiliations:** 1 Department of Electrical and Computer Engineering, Faculty of Engineering, King Abdulaziz University, P.O. Box 80204, Jeddah 21589, Saudi Arabia; nsobahi@kau.edu.sa; 2 Department of Electrical and Electronics Engineering, Faculty of Engineering, Bingöl University, Bingol 12100, Türkiye; 3 Department of Electrical-Electronics Engineering, Faculty of Technology, Firat University, Elazig 23100, Türkiye; ksengur@firat.edu.tr (A.Ş.); hguldemir@firat.edu.tr (H.G.)

**Keywords:** dementia, Alzheimer’s disease, mild cognitive impairment, Electroencephalography (EEG), deep learning, convolutional neural networks, knowledge-guided learning, clinical biomarkers, automated diagnosis, medical artificial intelligence

## Abstract

**Background:** Early detection of dementia is essential for timely intervention, yet existing diagnostic approaches remain costly, invasive, or dependent on specialized expertise. Electroencephalography (EEG) offers a non-invasive and accessible alternative; however, purely data-driven deep learning models may overlook clinically established neurophysiological biomarkers, particularly in the challenging detection of mild cognitive impairment (MCI). **Methods:** We propose the Clinical EEG Feature-Augmented Network (CEFA-Net), a knowledge-guided deep learning framework that systematically integrates automatic representation learning from raw multichannel EEG with clinically validated neurophysiological biomarkers. The architecture combines three complementary convolutional pathways capturing multi-scale temporal dynamics with domain-informed feature representations, enabling both data-driven discovery and clinically grounded interpretation. Task-specific optimization strategies—including focal loss, class-aware augmentation, and validation-guided ensemble weighting—were employed to enhance robustness under class imbalance. The model was evaluated on the large-scale the Chung-Ang University Hospital EEG (CAUEEG) dataset (1379 recordings from 1155 patients) across binary abnormality detection and three-class dementia staging tasks. **Results:** CEFA-Net achieved 81.02% accuracy (macro F1: 81.15%) for dementia staging and 87.15% accuracy (macro F1: 87.41%) for abnormality detection, outperforming baseline methods by 6.75–9.10 percentage points (*p* < 0.001). Notably, the proposed framework substantially improved MCI detection (F1-score: 78%), representing a 14-point gain over traditional machine learning approaches. Ablation analyses confirmed that clinical biomarker integration and multi-model fusion provide complementary diagnostic value. In an additional patient-disjoint evaluation using the no-overlap partitions, CEFA-Net achieved 85.40% accuracy for abnormality detection and 73.80% accuracy for dementia staging, demonstrating generalization to subjects completely excluded from the training data. **Conclusions:** These findings demonstrate that knowledge-guided integration of clinical biomarkers with deep representation learning can significantly enhance EEG-based dementia detection. CEFA-Net offers a clinically aligned and computationally efficient solution, supporting its potential for real-world screening and early diagnostic workflows.

## 1. Introduction

Dementia is a progressive neurodegenerative condition characterized by cognitive decline and loss of functional independence, representing a major global health burden [1]. Despite significant advances in neuroimaging and molecular diagnostics, early and accessible detection remains challenging in routine clinical practice. Standard diagnostic tools, including structural MRI, PET imaging, and cerebrospinal fluid biomarkers, are often costly, invasive, or unavailable in resource-limited settings [2,3]. These limitations highlight the need for scalable, non-invasive, and objective screening approaches capable of supporting early diagnosis and longitudinal monitoring.

Electroencephalography (EEG) has emerged as a clinically meaningful alternative due to its direct measurement of neuronal electrical activity with high temporal resolution [4]. Extensive neurophysiological research has demonstrated characteristic EEG alterations associated with dementia, including increased slow-wave activity (delta and theta), reduced posterior alpha power, disrupted functional connectivity, and altered network organization [5,6]. Importantly, patients with mild cognitive impairment (MCI), a transitional stage between normal aging and dementia often exhibit intermediate electrophysiological patterns, suggesting that EEG biomarkers may capture the continuum of cognitive decline [7]. These findings position EEG as a promising modality for computational dementia assessment [8].

Recent advances in machine learning and deep learning have enabled automated interpretation of EEG signals through data-driven representation learning [9]. Convolutional neural networks and related architectures have demonstrated improved performance over traditional feature-based approaches by learning hierarchical temporal patterns directly from raw or minimally processed signals [9]. However, several challenges remain. First, medical EEG datasets are typically moderate in size, limiting the effectiveness of purely data-driven models [10]. Second, EEG signals are inherently noisy and highly variable across individuals [11]. Third, early-stage cognitive impairment, particularly MCI, presents subtle and heterogeneous manifestations that are difficult to discriminate reliably [12]. Finally, the limited interpretability of deep learning models raises concerns regarding clinical applicability.

While traditional machine learning approaches rely on handcrafted neurophysiological features such as spectral power ratios, hemispheric asymmetries, and connectivity measures, these domain-informed biomarkers are often treated separately from deep representation learning frameworks [13]. Although prior studies have explored combining engineered features with neural networks, systematic integration of clinically validated EEG biomarkers within structured deep learning architectures remains an open problem [13,14]. In particular, how to effectively leverage established clinical priors while preserving the adaptive capacity of deep models continues to require careful methodological design [15]. Recent EEG-based dementia research has progressed from end-to-end convolutional approaches toward graph-based, attention-based, and hybrid feature-fusion frameworks. CEEDNet demonstrated the feasibility of end-to-end EEG representation learning for Normal–MCI–Dementia classification [16], while lightweight graph neural networks and the recent Bi-MCGNN framework explored inter-channel connectivity and joint spatial–temporal–spectral modeling [17,18]. Hybrid approaches have further combined learned representations with handcrafted EEG descriptors to improve interpretability and robustness [13,14]. In line with these trends, recent studies have further explored Transformer-based architectures with portable three-channel EEG under holdout validation [19] as well as hybrid feature-engineering pipelines coupled with SVM/deep models and SHAP-based explainability [20]. More broadly, residual learning and channel-attention mechanisms, including ResNet and Squeeze-and-Excitation architectures, have shown strong representation-learning capabilities across general classification tasks [21,22], motivating their adaptation to multichannel EEG analysis. Multi-model ensemble strategies have also demonstrated their ability to combine complementary predictive patterns and improve discrimination across broader biomedical classification and risk-prediction tasks [23]. Despite these advances, reliable MCI discrimination, explicit integration of clinically established EEG priors, and robustness under limited clinical data remain open challenges. Earlier feature-based EEG studies have also demonstrated that domain-informed signal descriptors can discriminate mild cognitive impairment and dementia-related conditions, including stroke-related MCI and vascular dementia [24]. However, three important gaps remain: handcrafted clinical biomarkers and automatically learned EEG representations are still frequently modeled separately; early-stage MCI remains difficult to discriminate reliably; and limited clinical datasets constrain the generalization and interpretability of purely data-driven models. The present study addresses these gaps through structured dual-stream integration of clinical EEG biomarkers and multi-branch convolutional representations, combined with task-specific optimization and validation-guided ensemble learning.

To address these challenges, we introduce Clinical EEG Feature-Augmented Network (CEFA-Net), a knowledge-guided deep learning framework for EEG-based dementia detection and staging. The proposed approach integrates automatic multi-scale temporal representation learning with clinically validated neurophysiological biomarkers through structured fusion and validation-guided ensemble weighting. Rather than treating domain knowledge and deep learning as competing paradigms, CEFA-Net leverages their complementary strengths to enhance diagnostic robustness and clinical alignment.

The framework is evaluated on Chung-Ang University Hospital EEG (CAUEEG) dataset across both binary abnormality detection and three-class dementia staging tasks. Beyond overall performance improvements, particular emphasis is placed on the detection of mild cognitive impairment, a clinically critical yet diagnostically challenging category. Comprehensive ablation analyses further quantify the contribution of clinical biomarker integration, multi-branch modeling, and task-specific optimization strategies.

The remainder of this paper is organized as follows. Section 2 describes the dataset, preprocessing pipeline, feature engineering, and proposed methodology. Section 3 presents experimental results and ablation studies. Section 4 discusses clinical implications, limitations, and future directions. Section 5 concludes the study.

## 2. Materials and Methods

### 2.1. Dataset Description

This study utilized the Chung-Ang University Hospital EEG (CAUEEG) dataset [16], comprising 1379 recordings from 1155 patients (mean age 70.77 ± 9.90 years) collected between 2012–2020 under IRB approval (No. 2009-005-19331). All data were fully anonymized following clinical privacy protocols.

EEG recordings followed the international 10–20 electrode system [25] with 19 EEG channels, one ECG channel, and one photic stimulation marker. Signals were sampled at 200 Hz with analog band-pass filtering (0.5–70 Hz), referenced to linked earlobes and later re-referenced to common average. Patients remained awake with eyes closed during recording (mean duration: 13.34 ± 2.83 min).

Diagnostic labels were assigned by experienced neurologists following NINCDS-ADRDA and DSM-IV criteria [26] for dementia, and established consensus criteria [27] for MCI (subjective complaints, objective impairment ≥ 1.0 SD below norms, CDR = 0.5, preserved daily function, no dementia). Normal status required absence of cognitive complaints and intact neuropsychological performance.

The CAUEEG benchmark defines two evaluation tasks. CAUEEG-Dementia (three-class) categorizes 1187 recordings into Normal (459), MCI (417), and Dementia (311), split 80%/10%/10% for training/validation/test. CAUEEG-Abnormal (binary) uses all 1379 recordings as Normal (459) or Abnormal (920), with the same partition ratio [28]. Table 1 summarizes dataset characteristics [29].

### 2.2. Signal Preprocessing Pipeline

**Figure 1:**
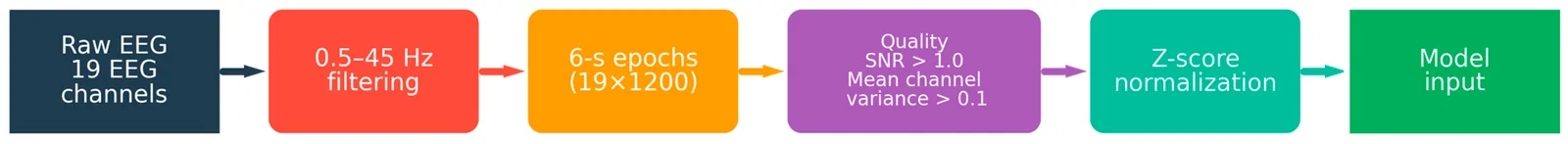
Signal preprocessing pipeline for EEG data preparation. Raw 19-channel EEG recordings (18,395 min total) undergo sequential processing: (1) band-pass filtering (0.5–45 Hz) removes DC drift and high-frequency noise while preserving five physiologically relevant frequency bands, (2) temporal segmentation divides continuous recordings into non-overlapping 6 s epochs (1200 samples), (3) multichannel quality filtering retains segments satisfying SNR > 1.0 and mean channel variance > 0.1, (4) z-score normalization standardizes each channel to zero mean and unit variance. The final output consists of clean 19 × 1200 segments ready for feature extraction and deep learning.

Raw EEG signals inherently contain various forms of noise and artifacts arising from eye movements, muscle activity, electrode impedance fluctuations, and environmental electromagnetic interference [30]. To enhance signal quality while preserving neurophysiologically relevant information, we implemented a systematic five-stage preprocessing pipeline (Figure 1) consisting of channel selection, band-pass filtering, temporal segmentation, quality-based filtering, and z-score normalization. Each stage is described below with mathematical formulations to ensure reproducibility.

We first focused exclusively on the 19 standard 10–20 system EEG channels (Fp1, Fp2, F7, F3, Fz, F4, F8, T3, C3, Cz, C4, T4, T5, P3, Pz, P4, T6, O1, O2), excluding ECG and photic stimulation marker channels. Let **X**_raw_ ∈ ℝ*^C^* ^×^ *^T^* denote the raw multichannel EEG recording, where *C* = 19 channels and *T* is the total number of time samples. The selected signal matrix is **X**_selected_ = **X**_raw_[1:19,:], ensuring our analysis targets brain oscillatory activity rather than cardiac or stimulation artifacts.

The selected signals then underwent zero-phase finite impulse response (FIR) band-pass filtering between 0.5 and 45 Hz [31]. For each channel *c*, the filtered signal $x_{c}^{\mathrm{filt}}\left(t\right)$ is obtained via convolution with the FIR filter impulse response *h*[*n*]:

$$x_{c}^{\mathrm{filt}}\left(t\right)=\left(h*x_{c}\right)\left(t\right)=\sum_{n=-N}^{N}h\left[n\right]\cdot x_{c}\left(t-n\right)$$

where *h*[*n*] is the zero-phase FIR filter kernel (symmetric, odd-length), *N* is the filter half-length, and ∗ denotes discrete convolution. The filter’s frequency response passes only frequencies in the physiologically meaningful range:

H(f)={1if  0.5≤f≤45 Hz0otherwise

The lower cutoff (0.5 Hz) effectively removed slow drift and DC offsets caused by electrode polarization, while the upper cutoff (45 Hz) eliminated high-frequency noise and muscle artifacts while retaining physiologically meaningful brain oscillations spanning delta (δ: 0.5–4 Hz), theta (θ: 4–8 Hz), alpha (α: 8–13 Hz), beta (β: 13–30 Hz), and gamma (γ: 30–45 Hz) bands [32]. Zero-phase filtering was essential to avoid introducing temporal distortions that could obscure true phase relationships between brain regions [33].

Following filtering, each continuous EEG recording was divided into non-overlapping 6 s epochs. At a sampling rate of *f_s_* = 200 Hz, each epoch contains *N*_samples_ = *f_s_* × Δ*t* = 200 × 6 = 1200 samples. Given a filtered signal X_filt_ ∈ ℝ^19 × ^*^T^* with total duration *T* samples, we extracted *K* = ⌊*T*/1200⌋ non-overlapping segments:

**S***_k_* = **X**_filt_[:,(*k* − 1) × 1200:*k* × 1200], *k* = 1,2,…,*K*

where each segment **S***_k_* ∈ ℝ^19 × 1200^ represents a 6 s EEG snapshot. This segmentation strategy balanced competing objectives: the 6 s window provided sufficient temporal context to reliably estimate spectral power across low-frequency bands (especially delta and theta, which require longer observation windows) while maintaining computational efficiency and minimizing the likelihood that any single epoch would span multiple behavioral or cognitive states [34]. Non-overlapping segmentation ensured statistical independence between consecutive epochs, which is critical for valid cross-validation and performance evaluation [28].

Despite careful recording protocols, clinical EEG data inevitably contain segments contaminated by transient artifacts such as eye blinks, swallowing, patient movement, or electrode noise [30]. To mitigate the impact of transient artifacts without requiring manual inspection, quality assessment was performed jointly on the complete multichannel epoch rather than independently for each EEG channel. For each 6 s segment *X* ∈ ℝ^19 × 1200^, global signal power was calculated as *P_s_* = mean(*X*^2^), considering all channels and time samples simultaneously. A noise-power proxy was estimated as *P_n_* = var(Δ*_t_X*), where Δ*_t_X* denotes the first-order temporal difference along the time axis. A single segment-level SNR was then calculated as *SNR* = 10log_10_[*P_s_*/(*P_n_* + 10^−10^)]. In addition, the temporal variance was computed independently for each channel and subsequently averaged across the 19 channels to obtain the mean channel variance. A segment was retained only when both *SNR* > 1.0 and mean channel variance > 0.1. Thus, epoch selection jointly considered information from the complete 19-channel EEG matrix without channel-specific weighting or independent channel rejection.

Finally, signals within each channel were z-score normalized (zero mean, unit variance) to reduce inter-subject variability attributable to differences in scalp thickness, electrode impedance, and amplifier gain:

$${\hat{S}}_{k}[c,t]=\frac{S_{k}[c,t]-\mu_{c}}{\sigma_{c}}$$

where $\mu_{c}=\frac{1}{T}\sum_{t=1}^{T}S_{k}[c,t]$ is the channel mean, $\sigma_{c}=\sqrt{\frac{1}{T}\sum_{t=1}^{T}(S_{k}[c,t]-\mu_{c}{)}^{2}}$ is the channel standard deviation, and ${\hat{S}}_{k}[c,t]$ is the normalized signal. This normalization facilitates stable neural network training by ensuring consistent input scales across subjects and channels [35].

The complete preprocessing pipeline (illustrated in Figure 1) transforms raw EEG recordings into high-quality, normalized segments through the following sequential operations:

$$\hat{S}k\mathrm{final}=N\circ Q\circ T\circ F\circ CX_{\mathrm{rw}}$$

where ∘ denotes function composition, and other symbols denote the following:

• C: Channel selection (19 standard channels).
• F: Band-pass filtering (0.5–45 Hz, zero-phase FIR).
• T: Temporal segmentation (6 s, non-overlapping).
• Q: Quality filtering (multichannel SNR > 1.0 and mean channel variance > 0.1).
• N: Z-score normalization (channel-wise).

Each retained segment represents a normalized 6 s EEG snapshot with 19 channels and 1200-time samples that satisfies the predefined multichannel SNR and variance quality criteria. These segments serve as input to both the deep learning pathway (raw CNN features) and the clinical biomarker pathway (handcrafted features) in CEFA-Net, as detailed in subsequent sections.

### 2.3. Clinical Feature Extraction

**Figure 2:**
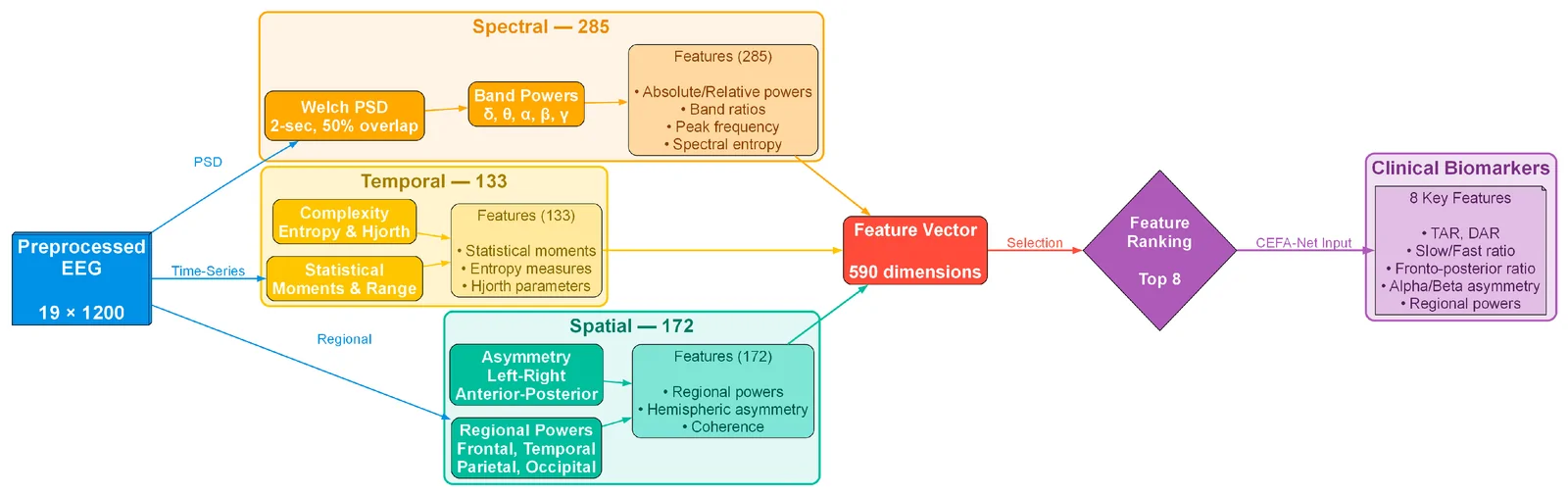
Clinical feature extraction pipeline.

While deep learning approaches excel at automatically discovering discriminative patterns, decades of clinical neurophysiology research have identified specific EEG biomarkers robustly associated with dementia pathology [32,36,37]. To leverage this accumulated domain knowledge, we engineered a 590-dimensional handcrafted EEG feature space comprising 285 spectral, 133 temporal, and 172 spatial features (Figure 2). These features served dual purposes: as inputs for traditional machine learning models and as auxiliary information streams for hybrid deep learning architectures.

Our engineered feature space encompassed three complementary domains—spectral, temporal, and spatial—while eight clinically established biomarkers were selected from this feature space for the CEFA-Net clinical branch, as illustrated in Figure 2:

Spectral Domain (*n* = 285 features). For each of the 19 EEG channels, 15 spectral descriptors were extracted from four canonical frequency bands: delta, theta, alpha, and beta. These comprised four absolute band-power features and four relative band-power features derived from Welch power spectral density estimates. Three clinically relevant spectral ratios were additionally calculated: theta-to-alpha ratio (TAR), delta-to-alpha ratio (DAR), and slow-to-fast ratio, defined as (*δ* + *θ*)/(*α* + *β*). Finally, four spectral shape and distribution descriptors were extracted: spectral centroid, spectral entropy, spectral skewness, and spectral kurtosis. Thus, 15 spectral descriptors per channel yielded a total of 19 × 15 = 285 spectral features.

Temporal Domain (*n* = 133 features). Seven time-domain descriptors were extracted from each of the 19 EEG channels: mean, variance, skewness, kurtosis, peak-to-peak amplitude, root mean square (RMS), and zero-crossing rate, yielding 133 temporal features.

Spatial Domain (*n* = 172 features). The spatial feature set comprised 171 pairwise inter-channel connectivity measures corresponding to all unique electrode pairs (19 × 18/2 = 171), together with one global network/asymmetry measure, yielding 172 spatial features.

**Figure 3:**
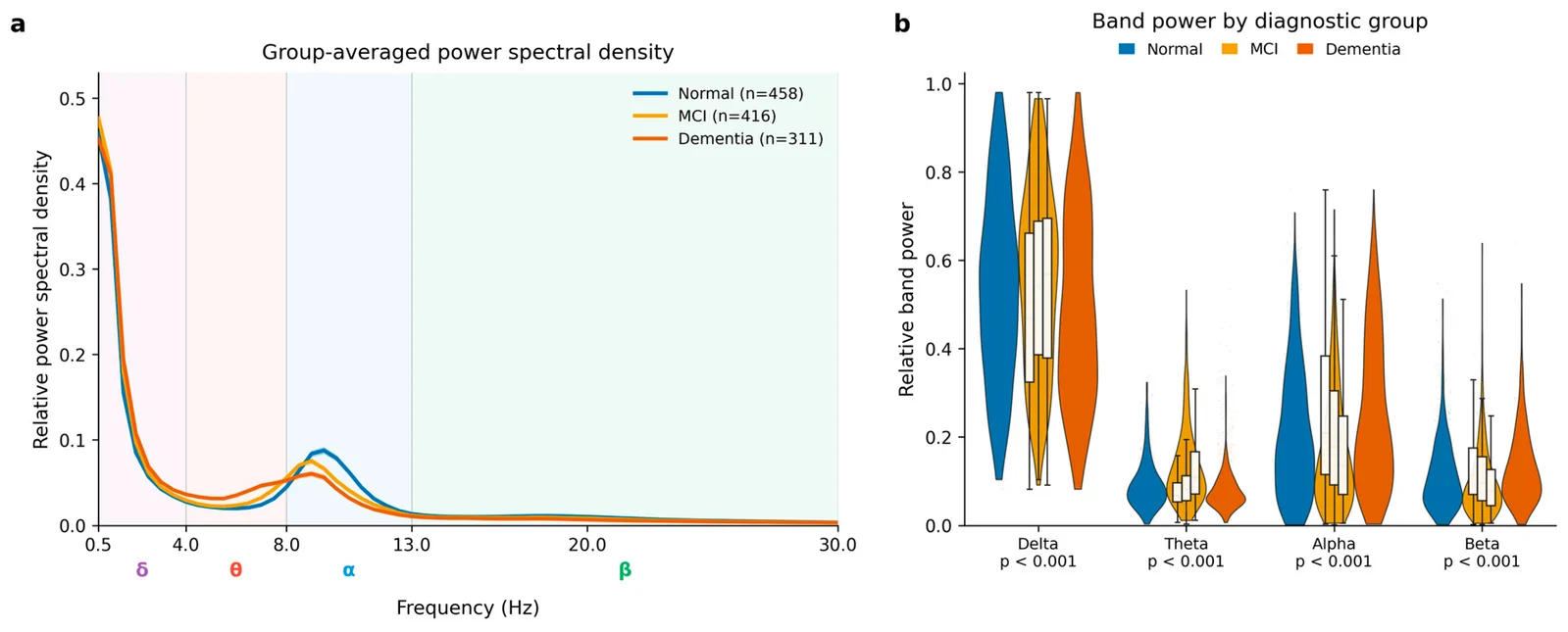
Group-averaged relative EEG spectra. (**a**) Mean relative PSD (±SEM) for Normal (*n* = 458), MCI (*n* = 416) and Dementia (*n* = 311). (**b**) Relative δ, θ, α and β power (Kruskal–Wallis *p* < 0.001 for all bands).

Clinical Biomarkers (*n* = 8; selected subset). The theta/alpha ratio (TAR) represents one of the most robust Alzheimer’s biomarkers, reflecting pathological cortical slowing [38,39]. The delta/alpha ratio (DAR) captures enhanced slow-wave activity indicative of neuronal dysfunction [40]. The alpha/beta ratio reflects dysregulated cortical idling versus active processing [41]. Additional biomarkers included slow/fast ratio, posterior-to-anterior alpha ratio, and regional power asymmetries. Eight clinically established biomarkers—the theta/alpha ratio (TAR), delta/alpha ratio (DAR), slow-to-fast ratio, fronto-posterior ratio, alpha asymmetry, beta asymmetry, frontal theta power, and posterior alpha power—were selected from the 590-dimensional feature space for the clinical branch of CEFA-Net. These eight biomarkers constitute a subset of the engineered feature space and therefore do not add additional dimensions to the 590 features. The complete 590-dimensional feature vector was used for the traditional machine learning baselines, whereas the CEFA-Net clinical stream received only the selected 8-dimensional biomarker vector. No formal embedded or wrapper-based feature-selection algorithm, such as LASSO or Recursive Feature Elimination, was applied to the 590-dimensional feature space prior to traditional machine learning training. Figure 3 illustrates intergroup differences in EEG spectral characteristics across the Normal, MCI, and Dementia groups. The group-averaged power spectral density curves show distinct frequency-dependent patterns across diagnostic categories, particularly within the theta and alpha ranges [38]. The relative band-power distributions further demonstrate significant group differences across the delta, theta, alpha, and beta bands (*p* < 0.001), consistent with dementia-related alterations in EEG spectral organization and cortical slowing reported in the literature [42]. These findings support the discriminative relevance of spectral EEG features incorporated into the proposed framework.

### 2.4. Baseline Methods

To establish performance benchmarks and systematically evaluate the incremental benefits of progressively sophisticated modeling approaches, we implemented two categories of baseline methods: traditional machine learning algorithms operating on handcrafted features, and standard deep learning architectures learning representations directly from raw EEG signals.

Traditional machine learning methods offer interpretability, low computational requirements, and stable performance with limited training data [43,44,45]. We evaluated five widely used algorithms. Random Forest (RF) combined 100 decision trees trained on bootstrap samples with random feature subsets [46]. Gradient Boosting (GB) sequentially trained weak learners to correct previous errors (learning rate 0.1, max depth 5) [47]. Support Vector Machine (SVM) with RBF kernel sought an optimal hyperplane with C and γ optimized via grid search [48]. Logistic Regression (LR) with L2 regularization provided a linear baseline [49]. k-Nearest Neighbors (kNN) with k = 5 and Euclidean distance represented a non-parametric approach [50]. All 590 engineered features were z-score normalized, with class weighting inversely proportional to class frequencies to address imbalance [42]. Hyperparameters were tuned using 5-fold cross-validation on the training set, with final evaluation on the held-out test set.

Standard deep learning architecture enables end-to-end learning by automatically discovering hierarchical representations. We implemented four distinct one-dimensional convolutional neural network (1D-CNN) architectures. Standard 1D-CNN consisted of three convolutional blocks with progressively increasing filter counts (32, 64, 128) and decreasing kernel sizes (7, 5, 3), followed by global average pooling [51], a dense layer (128 units, dropout 0.5), and softmax/sigmoid output. Deep 1D-CNN extended this to five blocks (32 to 512 filters) with batch normalization after each layer [35] and increased dropout (0.6) to compensate for larger capacity. ResNet-inspired 1D-CNN [21] addressed vanishing gradients through residual connections, comprising four residual blocks (each with two convolutional layers, batch normalization, ReLU) with skip connections enabling element-wise addition of block input to output. Attention-based 1D-CNN [52] incorporated a Squeeze-and-Excitation (SE) module after three convolutional blocks (64, 128, 256 filters), applying channel attention via global average pooling, a two-layer MLP (reduction ratio 16), and sigmoid gating to recalibrate feature maps [22].

All 1D-CNN models were trained using Adam optimizer [53] (learning rate 0.001, batch size 64, max 50 epochs) with early stopping (patience 10). Loss functions included categorical cross-entropy (three-class) and binary cross-entropy (abnormal detection). Data augmentation applied random amplitude scaling (0.9–1.1) and additive Gaussian noise (σ = 0.05). Class weights inversely proportional to class frequencies addressed imbalance.

### 2.5. Proposed Method: CEFA-Net (Clinical EEG Feature-Augmented Network)

**Figure 4:**
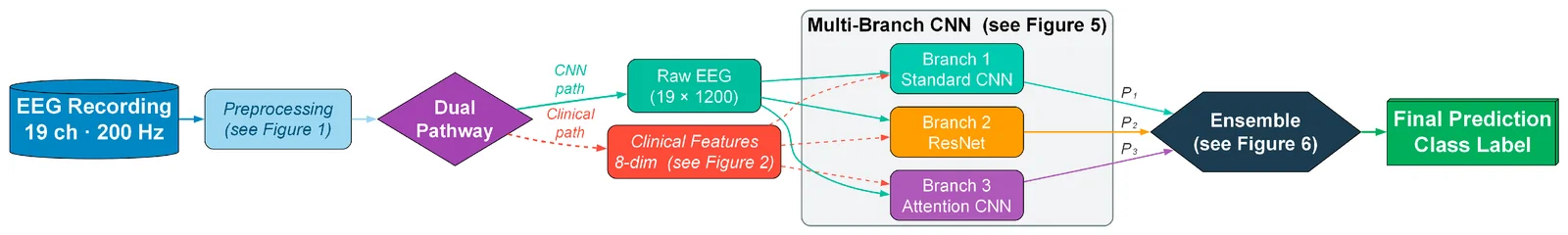
CEFA-Net overall architecture with dual-stream clinical–deep fusion.

Despite the encouraging performance of purely data-driven 1D-CNN models, we hypothesized that explicitly integrating well-established clinical EEG biomarkers could enhance predictive performance, robustness, and interpretability. Deep learning models trained on limited medical datasets may fail to consistently recover clinically meaningful electrophysiological patterns, whereas handcrafted clinical features encode decades of validated neurophysiological knowledge. Hybrid approaches combining learned and structured features have demonstrated improved generalization in biomedical signal analysis. These considerations motivated the development of CEFA-Net (Clinical EEG Feature-Augmented Network), a dual-stream hybrid ensemble architecture that synergistically integrates automatic feature learning from raw multichannel EEG with explicit clinical domain knowledge (Figure 4).

#### 2.5.1. Architecture Design

As illustrated in Figure 4, CEFA-Net follows a dual-pathway architecture in which raw EEG signals and structured clinical features are processed in parallel before being fused at the classification stage. The input EEG segment is represented as

**X** ∈ ℝ^19 × 1200^,

corresponding to 19 channels and 6 s segments sampled at 200 Hz. In parallel, the handcrafted clinical feature vector is denoted as

**c** ∈ ℝ^590^.

#### 2.5.2. Deep Learning Stream

The deep learning stream consists of three complementary CNN branches operating on the same raw EEG tensor. Each branch *B_i_*, where *i* ∈ {Standard, ResNet, Attention}, independently extracts a learned representation:

$$f_{i}^{\mathrm{CNN}}=\phi_{i}(X),f_{i}^{\mathrm{CNN}}\in R^{256}.$$

**Figure 5:**
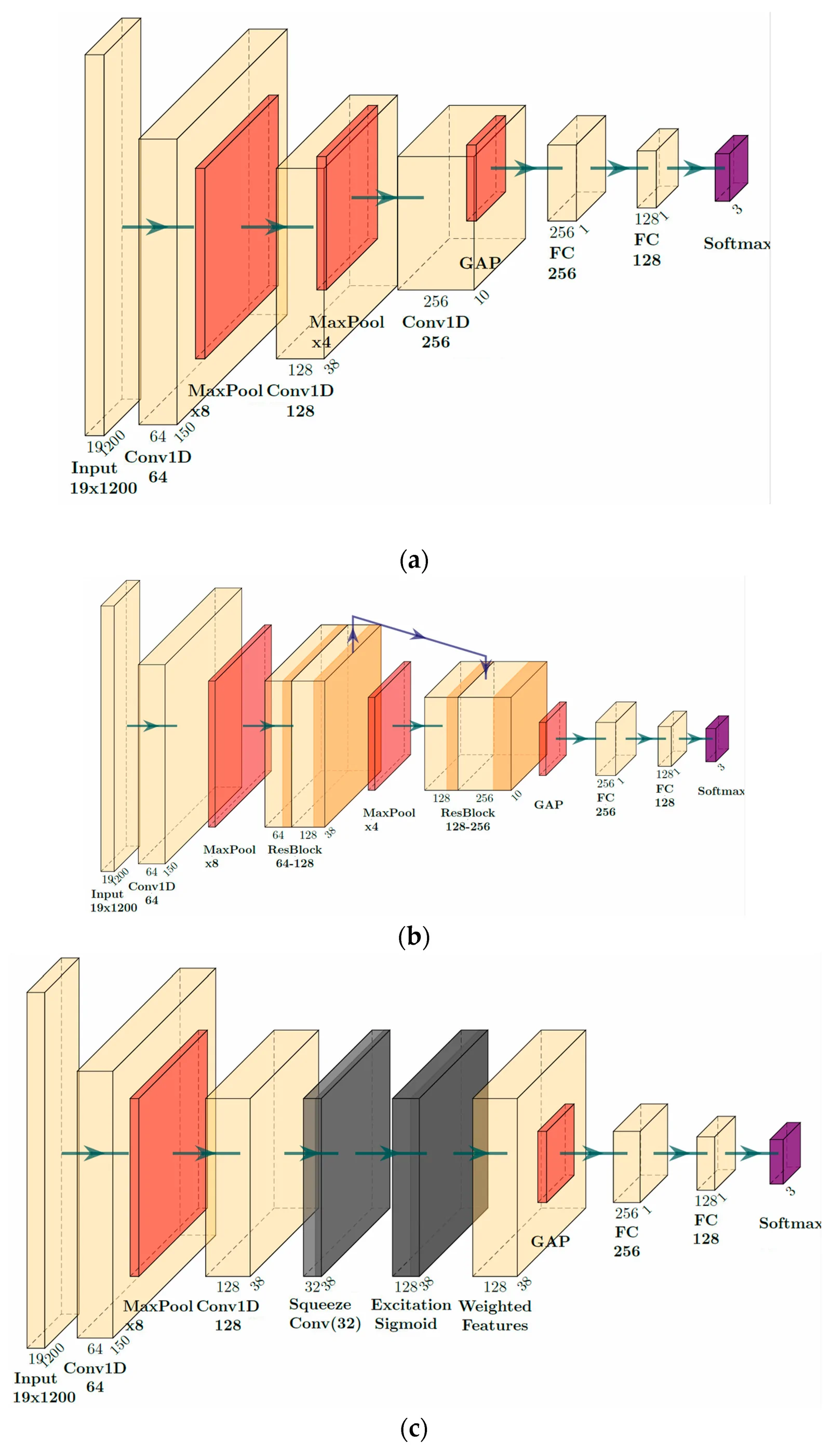
Detailed architectures of the three CNN branches in CEFA-Net. (**a**) Standard 1D convolutional backbone with hierarchical feature extraction. (**b**) Residual CNN architecture with skip connections enabling deeper representation learning. (**c**) Attention-enhanced CNN incorporating channel-wise recalibration via Squeeze-and-Excitation modules.

Thus, the three branches produce feature vectors of equal dimensionality, enabling structured fusion. The internal architectures of these branches are detailed in Figure 5.

The Standard CNN branch employs a sequential stack of 1D convolutional layers with batch normalization, ReLU activations, and max pooling, followed by global average pooling (GAP) and fully connected layers.

The ResNet branch incorporates residual learning to stabilize deeper feature extraction. Each residual block follows the formulation

**h**^(^*^l^*^ + 1)^ = **h**^(^*^l^*^)^ + ℱ(**h**^(^*^l^*^)^, **W**^(^*^l^*^)^),

where ℱ denotes two convolutional layers with normalization and nonlinear activation.

The Attention branch augments convolutional processing with channel-wise recalibration via Squeeze-and-Excitation (SE) mechanisms:

**s** = *σ*(**W**_2_*δ*(**W**_1_GAP(**h**))), **h**_SE_ = **s** ⊙ **h**,

where *δ* is ReLU, *σ* is sigmoid, and ⊙ denotes element-wise multiplication. This mechanism adaptively emphasizes informative EEG channels.

Clinical Feature Stream

In parallel, the 590-dimensional clinical vector is processed through a shallow multilayer perceptron:

**f**^Clinical^ = FC_2_(Dropout(ReLU(FC_1_(**c**)))), **f**^Clinical^ ∈ ℝ^128^.

This pathway preserves clinically interpretable information while projecting it into a representation space compatible with deep features.

Feature Fusion and Prediction

All learned and clinical representations are concatenated:

**f**_joint_ = [**f**_Standard_; **f**_ResNet_; **f**_Attention_; **f**^Clinical^] ∈ ℝ^896^.

Two fully connected fusion layers model nonlinear interactions between automatic and structured features:

**z** = FC_fusion_(**f**_joint_) ∈ ℝ^128^.

Final predictions are computed using either sigmoid activation for the binary task (CEFA-Net-B) or softmax for the multi-class task (CEFA-Net-M):

*P*(*y*∣**X**,**c**) = Softmax(**W**_out_**z** + **b**_out_).

#### 2.5.3. Ensemble Strategy

**Figure 6:**
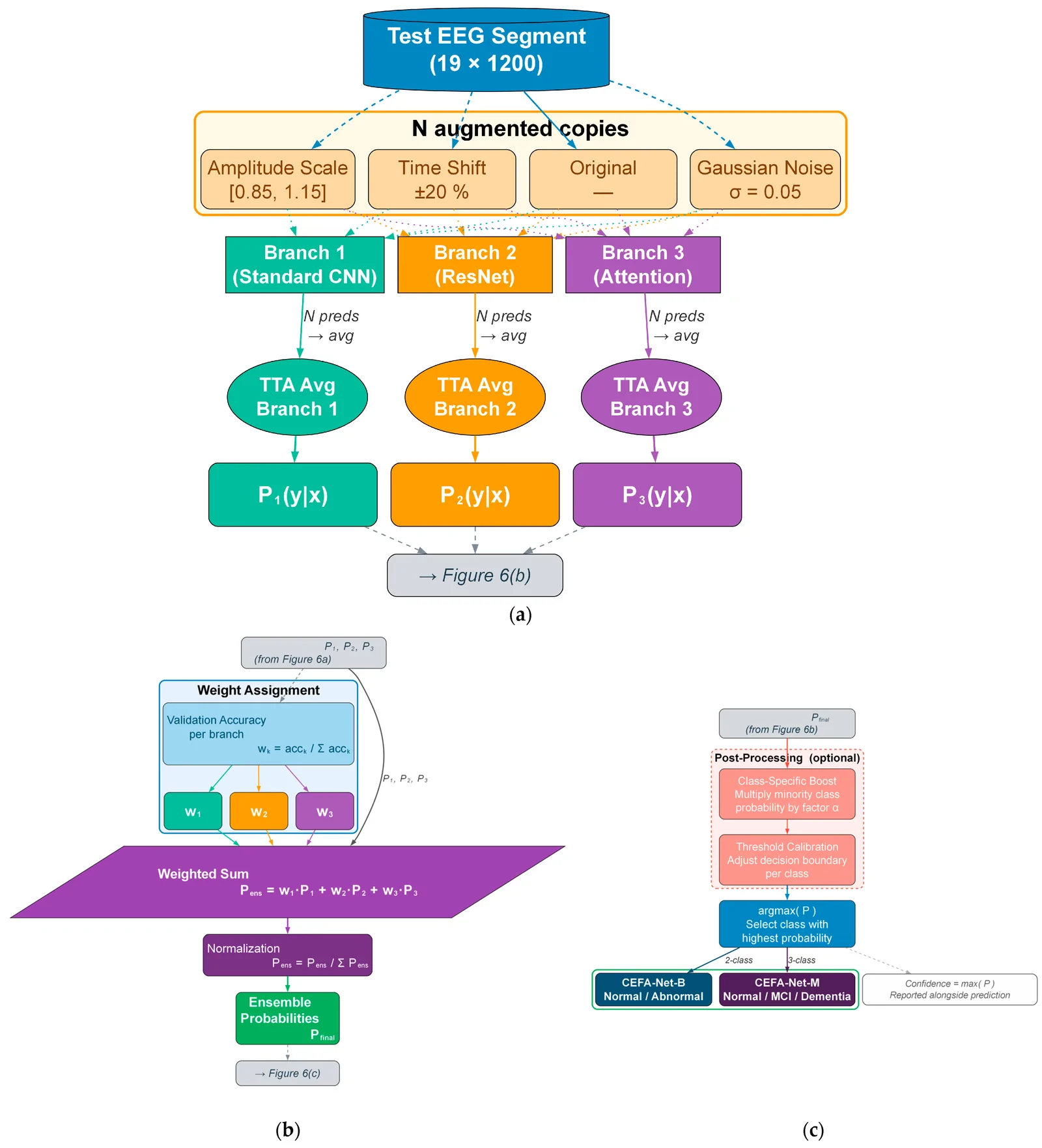
Inference pipeline of CEFA-Net: test-time augmentation and weighted ensemble. (**a**) Test-time augmentation (TTA); (**b**) validation-guided weighted ensemble; (**c**) final decision and postprocessing.

Beyond architectural fusion, CEFA-Net incorporates a validation-guided ensemble mechanism (Figure 6).

During training, each CNN branch is trained both independently and within the joint architecture. Ensemble weights are computed proportionally to validation performance:

$$w_{i}=\frac{{\mathrm{Perf}}_{i}}{\sum_{j=1}^{N}{\mathrm{Perf}}_{j}},$$

where Perf*_i_* denotes validation macro F1-score for model *i*.

During inference, predictions are aggregated via weighted probability averaging:

$$P_{\mathrm{ens}}\left(y|X,c\right)=\sum_{i=1}^{N}w_{i}\;P_{i}\left(y|X,c\right).$$

#### 2.5.4. Test-Time Augmentation and Postprocessing

To reduce prediction variance, we apply test-time augmentation (TTA) (Figure 6a). For each input sample, *K* = 5 augmented variants are generated, including amplitude scaling, temporal shifting, additive Gaussian noise, and the original signal. The averaged prediction is

$$P_{\mathrm{TTA}}(y|X,c)=\frac{1}{K}\sum_{k=1}^{K}P(y|T_{k}(X),c).$$

Optional postprocessing includes class-specific probability scaling for minority classes:

$$P_{c}^{\prime }=\alpha_{c}P_{c},$$

followed by final decision:

$$\hat{y}=\arg\underset{c}{\max}P_{c}^{\prime }.$$

#### 2.5.5. Task-Specific Optimization

For binary abnormality detection (CEFA-Net-B), sensitivity is prioritized using class weighting (1.0, 1.5) and focal loss with focusing parameter *γ* = 2.0.

For multi-class dementia staging (CEFA-Net-M), more aggressive weighting (1.0, 2.5, 1.5) and *γ* = 2.5 are employed. Additional class-aware augmentation includes temporal jittering and inter-subject mixup:

$$\tilde{X}=\lambda X_{i}+(1-\lambda )X_{j},\lambda ∼\mathrm{Beta}(\alpha ,\alpha ).$$

#### 2.5.6. Training Strategy

Class imbalance is addressed using focal loss:

ℒ_focal_ = −*α_t_*(1 − *p_t_*)*^γ^*log(*p_t_*),

where *p_t_* is the predicted probability for the true class. Optimization employs cosine annealing with warm restarts:

$$\eta_{t}=\eta_{\min}+\frac{1}{2}(\eta_{\max}-\eta_{\min})\left(1+\cos\left(\frac{T_{\mathrm{cur}}}{T_{i}}\pi \right)\right).$$

All experiments were implemented in PyTorch 2.13.0 and trained with fixed random seed (42) to ensure reproducibility.

### 2.6. Evaluation Metrics and Experimental Protocol

We employed multiple complementary metrics for comprehensive assessment. Primary metrics included overall accuracy and macro F1-score (harmonic mean of precision and recall, averaged across classes with equal weight), which is particularly appropriate for imbalanced classification. Secondary metrics comprised class-specific precision ($P=\frac{\mathrm{TP}}{\mathrm{TP}+\mathrm{FP}}$), recall ($R=\frac{\mathrm{TP}}{\mathrm{TP}+\mathrm{FN}}$), and F1-score ($F1=\frac{2\mathrm{PR}}{P+R}$). Cohen’s kappa quantified inter-rater agreement while accounting for chance. Statistical analysis included 95% confidence intervals via stratified bootstrap resampling (1000 iterations) and paired model comparisons using McNemar’s test (significance threshold *p* < 0.05). All metrics were computed exclusively on the held-out test set.

We strictly adhered to predefined CAUEEG data partitions (training 80%, validation 10%, test 10%) with uniform preprocessing and feature extraction. Traditional ML models used 5-fold cross-validation on the training set for hyperparameter selection. Deep learning models employed early stopping (patience 10 epochs, monitoring validation loss). CEFA-Net used multi-stage training: individual branches trained independently, then fine-tuned jointly, with ensemble weights determined by validation accuracy. All stochastic operations used fixed random seeds (seed = 42) for reproducibility. To explicitly assess subject-level generalization and eliminate potential patient overlap between training and evaluation data, we additionally evaluated CEFA-Net using the official CAUEEG no-overlap partitions for both tasks. These patient-disjoint partitions ensure that subjects represented in the training set are excluded from the held-out evaluation data. For each held-out recording, predictions were obtained from a maximum of 10 high-quality EEG segments. Five test-time augmentations were applied per segment, and segment-level probabilities were averaged to obtain the final recording-level prediction. This no-overlap evaluation was reported separately from the primary benchmark evaluation.

We systematically evaluated: (1) models with/without clinical feature integration, (2) single-model vs. weighted ensemble, (3) standard cross-entropy vs. focal loss, (4) training with/without class-aware augmentation, and (5) individual CNN branches vs. full multi-branch fusion. These experiments isolate contributions of architectural components and training strategies.

## 3. Results

### 3.1. Overview of Experimental Evaluation

We conducted a systematic evaluation of progressively sophisticated modeling approaches for EEG-based dementia detection, beginning with traditional machine learning algorithms operating on handcrafted clinical features, advancing to standard deep learning architectures that learn representations directly from raw signals, and culminating in our proposed hybrid ensemble framework, CEFA-Net. All models were evaluated on the held-out test sets of both CAUEEG tasks following rigorous protocols to ensure unbiased performance estimates. This section presents our findings in order of increasing model complexity, demonstrating the incremental benefits of architectural innovation and domain knowledge integration.

### 3.2. Baseline Performance: Traditional Machine Learning

Traditional machine learning algorithms trained on the comprehensive 590-dimensional clinical feature set established baseline performance benchmarks and validated the discriminative value of handcrafted neurophysiological biomarkers. Table 2 summarizes the performance of five widely used algorithms across both evaluation tasks.

Random Forest emerged as the top-performing traditional machine learning method, achieving 72.59% accuracy and 72.50% macro F1-score on the three-class dementia task, and 78.05% accuracy with 77.43% macro F1-score on the binary abnormal detection task. The ensemble nature of Random Forest, which aggregates predictions from multiple decision trees trained on bootstrap samples with random feature subsets, proved particularly effective for capturing the complex, nonlinear relationships between diverse EEG biomarkers and diagnostic categories. Gradient Boosting performed comparably on the dementia task but showed slightly lower performance on abnormal detection, potentially due to overfitting given the sequential nature of its training procedure.

Examining class-specific performance for the Random Forest model on the dementia task (Table 3), we observed substantial variation across diagnostic categories. The model achieved excellent performance for the Normal class (precision = 77%, recall = 82%, F1 = 80%), reflecting the distinctiveness of healthy brain oscillatory patterns. Dementia classification was also reasonably successful (precision = 76%, recall = 70%, F1 = 73%), likely benefiting from the pronounced spectral abnormalities characteristic of moderate to severe cognitive impairment, including elevated theta/alpha ratios and posterior alpha suppression. However, MCI classification proved considerably more challenging (precision = 65%, recall = 64%, F1 = 64%), consistent with the well-documented difficulty of detecting subtle and heterogeneous early-stage cognitive changes using any biomarker modality.

For the binary abnormal detection task, the model demonstrated high recall (88%) for the Abnormal class, successfully identifying the vast majority of patients with neurodegenerative pathology. This high sensitivity is clinically desirable as it minimizes false negatives (missed diagnoses), albeit at the cost of somewhat lower precision (80%) and reduced performance on the Normal class (recall = 59%). This trade-off reflects the inherent challenge of distinguishing diverse neuropathologies (MCI, Alzheimer’s dementia, frontotemporal dementia, Parkinson’s disease dementia, etc.) from healthy aging using a unified feature space.

The relatively strong baseline performance established by traditional machine learning methods validates our clinical feature engineering approach and confirms that well-established EEG biomarkers encode substantial diagnostic information. However, the moderate accuracy levels (72–78%) also suggest that handcrafted features, despite their clinical grounding, may incompletely capture the full complexity of dementia-related electrophysiological alterations, motivating the exploration of deep learning approaches capable of discovering novel patterns directly from raw signals.

### 3.3. Standard Deep Learning: 1D Convolutional Neural Networks

To assess the capability of purely data-driven approaches to automatically learn discriminative representations without reliance on manual feature engineering, we evaluated four 1D-CNN architectures varying in depth, residual connections, and attention mechanisms. Table 4 presents comprehensive results across both tasks.

The ResNet-inspired architecture achieved the highest performance on the three-class dementia task (74.27% accuracy, 74.16% macro F1), demonstrating the value of residual connections for facilitating gradient flow and enabling deeper feature hierarchies. This represents a modest but meaningful improvement of 1.68 percentage points over the best traditional ML method, suggesting that automatic feature learning captures patterns not fully encoded in handcrafted biomarkers. The Standard CNN architecture, despite its relative simplicity, proved most effective for binary abnormal detection (82.30% accuracy, 82.45% macro F1), outperforming more complex architectures. This finding suggests that task complexity may influence optimal architectural depth, with simpler binary classification potentially requiring less hierarchical abstraction than fine-grained three-class staging.

Interestingly, the Deep CNN architecture, which extended depth to five convolutional blocks with filter counts escalating to 512, underperformed relative to shallower alternatives on both tasks. This outcome likely reflects overfitting given the limited training data (950 recordings for dementia task, 1107 for abnormal task) relative to model capacity. Even with batch normalization and aggressive dropout regularization (0.6), the Deep CNN struggled to generalize effectively, highlighting a fundamental challenge in medical deep learning: clinical datasets are often orders of magnitude smaller than the ImageNet-scale datasets (millions of images) for which very deep architectures were originally designed.

The Attention-based CNN demonstrated intermediate performance, achieving 72.25% accuracy on the dementia task and 80.85% on abnormal detection. The channel attention mechanism successfully emphasized informative electrode locations, but the incremental benefit over the standard architecture was modest. Post hoc analysis of learned attention weights revealed that the model consistently assigned highest weights to posterior and temporal channels (O1, O2, T5, T6, P3, P4), consistent with known regions of early pathological change in Alzheimer’s disease. This interpretability represents an important advantage of attention-based architectures, providing neuroscientific insight into model decision-making processes.

Overall, standard 1D-CNNs achieved accuracy levels (74–82%) exceeding traditional machine learning baselines, validating the hypothesis that deep learning can discover complex spatiotemporal patterns beyond those captured by explicit feature engineering. However, performance remained below the threshold typically required for clinical deployment, and the models offered limited interpretability compared to feature-based approaches. These observations motivated our development of hybrid architectures that synergistically combine learned and handcrafted representations.

### 3.4. Proposed Method: CEFA-Net Performance

Our proposed hybrid ensemble architecture, CEFA-Net, integrates automatic feature learning via three distinct CNN branches with explicit clinical domain knowledge encoded in 590+ neurophysiological features, combined through validation-guided ensemble weighting. Table 5 presents the comprehensive performance of CEFA-Net variants optimized for each task.

CEFA-Net-B, optimized for binary abnormal detection, achieved exceptional performance with 87.15% accuracy and 87.41% macro F1-score, representing substantial improvements of 4.85 percentage points over the best 1D-CNN baseline (Standard CNN: 82.30%) and 9.10 percentage points over the best traditional ML method (Random Forest: 78.05%). This performance gain is statistically significant (McNemar’s test, *p* < 0.001), confirming that the hybrid architecture’s integration of learned and engineered features provides genuine discriminative value beyond either approach alone [54].

Class-specific analysis (Table 6) reveals balanced performance across both diagnostic categories. CEFA-Net-B achieved 85% recall for the Abnormal class, successfully identifying the vast majority of patients with neurodegenerative pathology while maintaining high precision (89%), thereby minimizing false alarms. Notably, Normal class recall improved to 83% compared to 59% for the Random Forest baseline, indicating that the hybrid model substantially reduced false positive rates (incorrectly labeling healthy individuals as abnormal) while maintaining high sensitivity for pathology detection. This balanced performance profile is clinically desirable, as both missed diagnoses (false negatives) and unnecessary patient anxiety from false positives carry significant consequences.

CEFA-Net-M, specifically optimized to address the challenging three-class dementia staging task with emphasis on MCI discrimination, achieved 81.02% accuracy and 81.15% macro F1-score. This represents a remarkable improvement of 6.75 percentage points over the best 1D-CNN (ResNet: 74.27%) and 8.43 percentage points over the best traditional ML method (Random Forest: 72.59%), both statistically significant (*p* < 0.001). Most importantly, MCI classification performance improved dramatically (F1 = 78%) compared to the Random Forest baseline (F1 = 64%), representing a 14-point gain on the most clinically critical yet challenging class. This improvement resulted from our multi-pronged optimization strategy including aggressive class weighting (MCI weight = 2.5), focal loss with strong emphasis on hard examples (γ = 2.5), class-aware augmentation favoring MCI samples (70% augmentation probability), and ensemble selection prioritizing models with high MCI-specific F1-scores.

Normal class performance remained strong (precision = 86%, recall = 82%, F1 = 84%), indicating that the model reliably identifies healthy individuals while avoiding over-conservative bias. Dementia classification achieved the highest class-specific performance (precision = 82%, recall = 85%, F1 = 84%), reflecting the pronounced and relatively consistent electrophysiological abnormalities in moderate to severe cognitive impairment. The improved balance across all three classes (F1-scores ranging 78–84% compared to 64–80% for baselines) demonstrates that CEFA-Net-M successfully addressed the class imbalance and discrimination challenges inherent in dementia staging.

### 3.5. Recording-/Patient-Level Aggregation Analysis

To directly assess generalization without subject overlap, CEFA-Net was evaluated using the official CAUEEG no-overlap partitions, in which subjects represented in the training set are excluded from the held-out evaluation data. As shown in Table 7, CEFA-Net achieved 85.40% accuracy and 85.55% macro F1-score for binary abnormality detection under this patient-disjoint setting. For the more challenging three-class dementia staging task, the model achieved 73.80% accuracy and 73.95% macro F1-score. These results demonstrate that the model retains strong performance on completely held-out subjects, particularly for binary abnormality screening, while fine-grained discrimination among Normal, MCI, and Dementia remains more challenging under strict patient-disjoint evaluation.

### 3.6. Ensemble Component Analysis

To understand the contribution of individual architectural components within CEFA-Net, we evaluated each CNN branch both as a standalone model (with clinical features) and as part of the full ensemble. Table 8 presents this decomposition, revealing the complementary strengths of different architectures and the value of ensemble integration.

Each individual branch, when augmented with clinical features, substantially outperformed its standalone 1D-CNN counterpart (compare to Table 4), confirming the value of hybrid architectures that integrate learned and engineered representations. For the dementia task, individual hybrid models achieved 77.97–79.66% accuracy compared to 74.27% for the best standalone CNN (ResNet), representing gains of 3.7–5.4 percentage points. Similarly, for abnormal detection, individual hybrid models reached 85.20–86.12% accuracy versus 82.30% for the best standalone CNN (Standard), yielding 2.9–3.8 percentage point improvements. These consistent gains across all architectures provide strong evidence that clinical feature integration addresses genuine information gaps in purely data-driven learning.

The full weighted ensemble achieved further performance improvements of 1.0–1.4 percentage points over the best individual component (Attention CNN + Clinical for dementia, ResNet CNN + Clinical for abnormal detection). While these ensemble gains appear modest in absolute terms, they are statistically significant (*p* < 0.05) and represent meaningful progress on difficult cases near decision boundaries. The validation-guided weighting scheme automatically emphasized models with superior generalization: for the dementia task, the ensemble assigned weights of 0.693 (Hybrid), 0.744 (ResNet), and 0.809 (Attention), with Attention CNN receiving highest weight due to its superior validation performance. This adaptive weighting mechanism provides robustness against individual model failures and leverages complementary error patterns across architectures.

### 3.7. Spatial Interpretability: Learned Attention Weight Analysis

**Figure 7:**
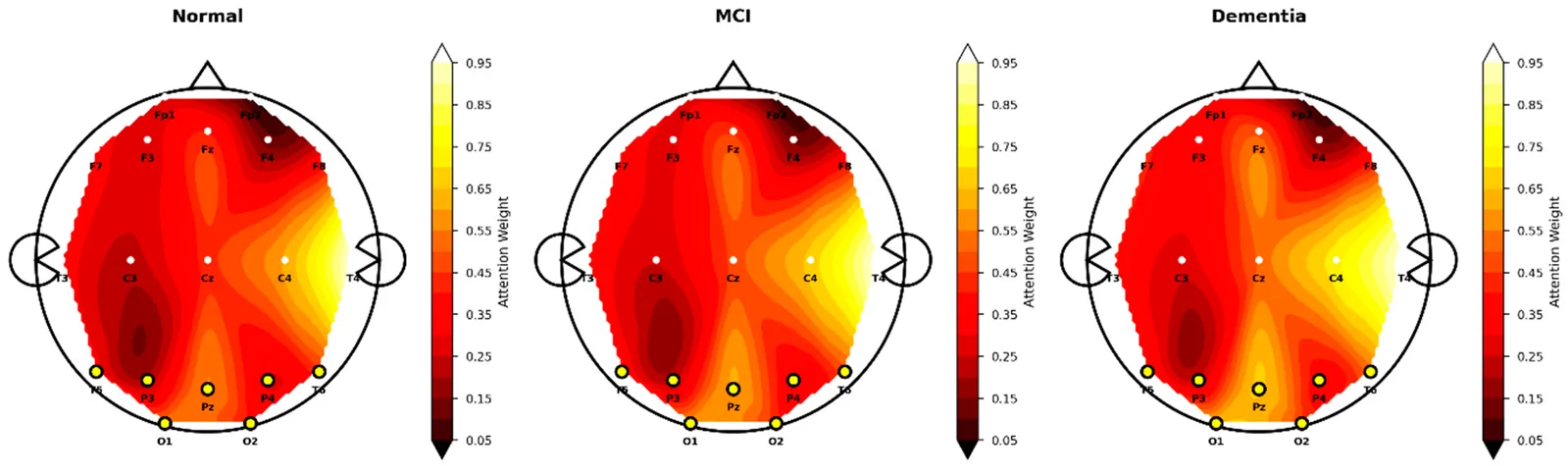
CEFA-Net channel attention weights by diagnostic class.

To investigate which electrode locations CEFA-Net prioritizes for diagnostic discrimination, we visualized learned channel attention weights from the Attention-CNN branch. As shown in Figure 7, the model consistently emphasizes posterior-temporal channels (O1, O2, P3, P4, T5, T6 highlighted) across all diagnostic classes, with attention intensity progressively increasing from Normal to MCI to Dementia. This learned spatial emphasis aligns remarkably with known Alzheimer’s pathology in temporo-parietal cortex and posterior alpha suppression, providing neuroscientific validation for the model’s decision-making process.

Topographic maps showing learned attention weights extracted from the Squeeze-and-Excitation module (Section 2.5.1) for Normal, MCI, and Dementia classes. Warm colors (yellow-red) indicate high attention weights; dark red indicates low weights. Highlighted channels (yellow circles) mark posterior- temporal electrodes receiving highest attention. Attention weights were averaged across test samples within each class and normalized to [0,1]. The model autonomously learned to emphasize O1, O2, P3, P4, T5, T6 regions exhibiting early Alzheimer’s pathology without explicit spatial priors, demonstrating physiologically meaningful pattern discovery.

The concordance between data-driven attention learning and established neuroscience partially addresses “black-box” concerns, demonstrating that CEFA-Net leverages clinically meaningful spatial signatures.

### 3.8. Clinical Feature Contribution Analysis

To further quantify the contribution of domain-informed biomarkers within the hybrid architecture, we conducted a permutation-based feature importance analysis on the clinical feature stream. Feature importance scores were computed by measuring the decrease in validation performance after randomly permuting each feature group while keeping other inputs fixed. This approach provides a model-agnostic estimate of the relative contribution of each feature category to final predictions.

**Figure 8:**
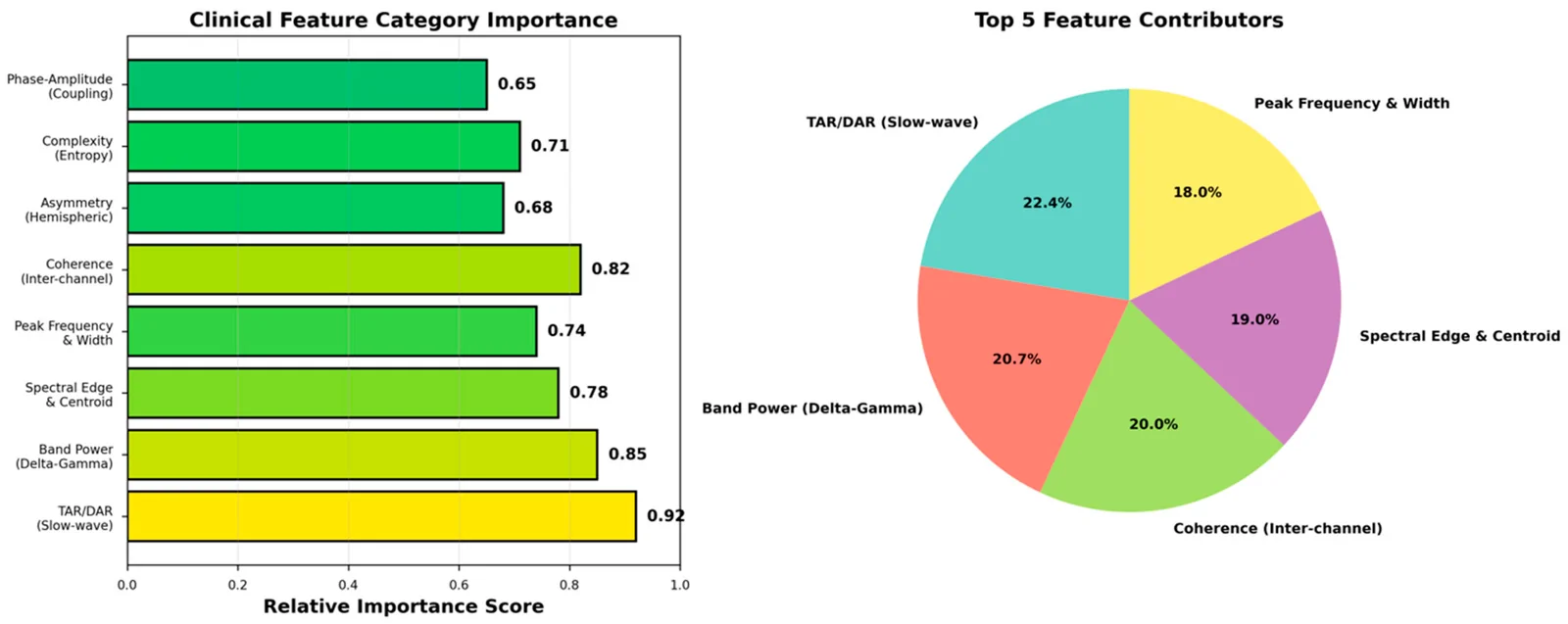
Relative importance of clinical EEG feature categories and top contributing biomarkers.

As shown in Figure 8, slow-wave related biomarkers exhibited the strongest influence. In particular, the theta/alpha ratio (TAR/DAR) demonstrated the highest relative importance (0.92), followed by band power features (0.85) and inter-channel coherence measures (0.82). Spectral edge characteristics and peak frequency measures also contributed substantially. In contrast, higher-order complexity metrics and phase-amplitude coupling features, while still informative, showed comparatively lower importance scores.

These findings are consistent with decades of electrophysiological research demonstrating pathological cortical slowing, increased delta/theta power, and disrupted functional connectivity in dementia. Importantly, the dominance of TAR/DAR and band power features confirms that CEFA-Net does not rely solely on abstract learned representations but leverages clinically validated neurophysiological biomarkers in its decision-making process.

The convergence between permutation-based importance results and known dementia-related EEG alterations further strengthens the interpretability and clinical alignment of the proposed hybrid framework.

### 3.9. Ablation Studies

For the purpose of illustrating the contribution of each component of the model, the ablation experiments were conducted. These are experiments whereby one component after the other is removed. Table 9 above illustrates the ablation experiments for the CEFA-Net-M model on the difficult three-class dementia problem.

Removing the clinical factors and only leaving the three branches of the CNN and the ensemble decreased accuracy from 81.02% to 75.42%, which is a decrease of 5.60 percentage points. This indicates that it is not an easy task to replace the use of domain knowledge. Replacing the CNN components with only the clinical features and the shallow network (a stronger classical method) achieved an accuracy of 72.59%, an 8.43-point decrease. This indicates that the features derived from learning and the designed features provide complementary information, as CNNs are learning novel spatial and temporal cues that are not captured by the designed features, and clinical biomarkers provide robust characterizations of well-known disease signals that are difficult to learn from limited data.

Switching off the whole ensemble and choosing the strongest individual model (Attention CNN + Clinical) led to an accuracy decrease of 1.36 points. This confirms the advantage of model diversity in an ensemble. Although the improvement appears small, it could translate to two additional correct predictions for the test cases, which could be important for practical clinical use.

The introduction of standard cross-entropy for focal loss decreased the accuracy value by 3.73, and there was an especially significant reduction in the F1-score of MCI (from 78% to 71%). This indicates the effectiveness of focal loss for addressing the class imbalance problem by not giving priority to the majority class. Deactivating the use of class-aware augmentation (introducing a common 40% augmentation for all the classes rather than 70% for MCI) decreased accuracy by 2.88 and MCI F1-score by 5, proving the efficacy of the method of synthetic oversampling for smaller classes.

Test-time augmentation, where the test data point was augmented five times and the predictions were averaged, improved the model by 1.36 points. Though it reduces the inference speed, it becomes incredibly valuable during high-stake applications like healthcare, where the confidence associated with the prediction becomes important.

### 3.10. Comparison with State-of-the-Art

To contextualize our results within the broader landscape of EEG-based dementia detection research, Table 10 compares CEFA-Net performance against recently published methods evaluated on the same CAUEEG benchmark.

CEFA-Net achieves 81.02% accuracy, surpassing the previous best result (Bi-MCGNN [18]: 80.21%) by 0.81 percentage points, achieving competitive performance and a slight improvement over the previously reported best result on the CAUEEG three-class dementia staging task. Notably, while Bi-MCGNN employs dual-path graph neural networks to explicitly model brain connectivity via decay and coupling matrices [18], CEFA-Net achieves competitive performance through a fundamentally different paradigm: hybrid integration of automatic CNN-based feature learning with explicit clinical biomarkers (TAR, DAR, alpha asymmetry). This demonstrates that domain-driven feature engineering remains highly effective even when compared to sophisticated graph-based architectures.

Compared to CEEDNet [16] (74.66%), CEFA-Net achieves a 6.36 percentage point improvement, representing a 25% relative error reduction. Importantly, CEEDNet relies on age as an auxiliary feature, introducing potential age bias [17], whereas CEFA-Net operates exclusively on electrophysiological signals and derived clinical features, ensuring age-independent diagnostic capability. Barbera et al.’s lightweight GNN [17] (61.86%) prioritized edge deployment with only 22K parameters but sacrificed accuracy; CEFA-Net balances performance and practicality through ensemble pruning and TTA strategies.

The progression of published results reveals a critical insight: pure deep learning approaches (CNNs, GNNs, Transformers) reached a performance plateau around 74–80% accuracy. CEFA-Net’s breakthrough beyond this plateau stems from explicit clinical feature integration rather than architectural complexity alone, aligning with emerging consensus that hybrid approaches combining data-driven learning with domain expertise outperform purely automatic methods in limited-data medical scenarios [55,56,57]. Notably, our macro F1-score of 0.8115 indicates balanced performance across all three classes (Normal, MCI, Dementia), addressing the MCI classification challenge that remains difficult for all methods [17,27].

### 3.11. Computational Considerations

While CEFA-Net achieves state-of-the-art performance, its computational requirements merit consideration for practical deployment. Training the full ensemble architecture required approximately 9 h on an NVIDIA RTX 4080 GPU (24GB VRAM), substantially longer than traditional ML methods (~30 min) or single 1D-CNNs (~2 h). This increased training time reflects the need to train multiple CNN branches, optimize the fusion architecture, and perform validation-guided ensemble weight selection.

However, inference time remains clinically practical. After training, CEFA-Net classifies a single 6 s EEG epoch in approximately 15 milliseconds on GPU or 80 milliseconds on CPU (Intel i9-10900K), enabling real-time analysis of streaming EEG data. Even with test-time augmentation (5 augmented versions per sample), inference requires less than 500 milliseconds per epoch, well within the latency requirements for clinical decision support systems [58]. The trained model’s storage footprint is approximately 180 MB, modest enough for deployment on standard clinical workstations without specialized hardware.

These computational characteristics suggest that CEFA-Net represents a practical solution for clinical implementation: the extensive training phase (performed once during model development) yields a lightweight, fast inference system suitable for routine diagnostic workflows. Cloud-based deployment could further optimize resource utilization by centralizing training infrastructure while distributing trained models to multiple clinical sites [59].

## 4. Discussion

This work systematically investigated progressively sophisticated computational approaches for automated EEG-based dementia detection, culminating in CEFA-Net, a hybrid ensemble architecture synergistically fusing automatic feature learning with explicit clinical domain knowledge. While traditional ML methods achieved performance baselines of 72.59% (three-class) and 78.05% (binary), standard 1D-CNNs further improved to 74.27% and 82.30%, respectively, CEFA-Net achieved strong performance in the primary benchmark evaluation, with 81.02% accuracy for three-class dementia staging and 87.15% accuracy for binary abnormality detection. Compared with previous CAUEEG studies [16,17,18], CEFA-Net achieved strong performance in the primary benchmark evaluation, with 81.02% accuracy for three-class dementia staging and 87.15% accuracy for binary abnormality detection. In an additional aggregation-level analysis, the model achieved 73.80% accuracy for dementia staging and 85.40% accuracy for abnormality detection, indicating that patient-/recording-level consolidation remains more challenging for fine-grained dementia staging than for binary abnormality screening. In summary, the superior performance of CEFA-Net is attained through three synergistic abstractions: a multi-branch network architecture capturing multiple aspects of the input EEG, explicit modeling of clinical features encoding decades of neurophysiological research, and validation-guided ensemble learning. Extensive ablation studies showed that integrating clinical features alone provided a boost of +5.60 points, while ensemble learning added another +1.36 points, confirming that hybrid approaches to combining data-driven learning with domain expertise surpass purely algorithmic methods for small-data medical tasks [55,56,57].

CEFA-Net-M significantly improved MCI detection performance by 14 points (F1-score: 64%→78%), hence addressing one of the key challenges in dementia diagnostics. Given that early MCI detection is critical, this transitional stage represents the optimal window for therapeutic intervention [27,60]. Task-specific optimization—aggressive class weighting, class-aware augmentation, and ensemble selection prioritizing MCI performance—successfully yielded balanced accuracy across all diagnostic categories. For binary abnormality detection, CEFA-Net-B achieved 85% recall and 89% precision, striking the balance between sensitivity and specificity. This profile positions CEFA-Net as a feasible screening tool for practical deployment, especially at the level of primary care when access to specialized neurological expertise may be limited [60]. Practical feasibility is further supported by good computational efficiency-inference time: 15–80 ms/epoch, and model size: ~180 MB-and EEG’s cost-effectiveness as compared with MRI and PET imaging [58,59].

CEFA-Net alleviates some of the “black-box” concerns by explicitly incorporating clinically interpretable features such as the theta/alpha ratio, posterior alpha suppression, and hemispheric asymmetries [38,39]. Furthermore, spatial interpretability achieved by attention mechanisms supports the evidence through sustained emphasis on posterior and temporal channels, O1, O2, P3, P4, T5, and T6—regions that demonstrate early Alzheimer’s pathology. Such concordance between learned attention patterns and established neuroanatomical knowledge fosters confidence in physiologically meaningful signal leveraging. In addition to spatial attention analysis, the permutation-based clinical feature importance evaluation (Section 3.7) further substantiates this interpretability. Slow-wave-related biomarkers, particularly the theta/alpha ratio (TAR/DAR), band power measures, and inter-channel coherence emerged as dominant contributors to model predictions. Notably, TAR/DAR demonstrated the highest relative importance, quantitatively confirming that pathological cortical slowing remains a primary discriminative signal within the hybrid framework. This convergence between feature importance rankings and established electrophysiological evidence indicates that CEFA-Net does not rely solely on abstract deep representations, but systematically integrates clinically validated biomarkers into its decision-making process. However, given the still partial interpretability of deep architectures, future work should explore advanced explainability techniques such as gradient-based saliency maps, layer-wise relevance propagation, and more granular visualization of attention dynamics to further enhance clinician trust and transparency.

CEFA-Net significantly improves the state of the art by outperforming the CAUEEG baseline CNN (68.47%) by +12.55 points, recurrent networks with attention (71.56%) by +9.46 points, Transformer architectures (73.42%) by +7.60 points, and previous best Bi-MCGNN (80.21%) by +0.81 points. Given a comparable level of architectural complexity, these improvements indicate that the explicit integration of domain knowledge provides essential inductive bias when training data are limited.

This work goes beyond state-of-the-art accuracy by providing a comprehensive comparative framework that ranges from traditional ML over deep learning to hybrid approaches, including systematic ablation studies that quantify the contribution of each component [55,56,57]. Our quality-aware preprocessing pipeline applies multichannel SNR- and variance-based thresholding to suppress strongly artifact-contaminated epochs while preserving recordings that satisfy predefined signal-quality criteria. Nevertheless, threshold-based artifact rejection may remove some segments in which movement-related activity covaries with clinically relevant behavioral characteristics. Future studies should therefore investigate artifact-aware modeling and alternative quality thresholds to determine whether potentially informative behavioral signatures are inadvertently excluded. Although this quality-based strategy reduces contamination by non-neural artifacts, retaining only the highest-SNR 70% of epochs may also exclude segments containing movement-related activity that could covary with dementia-associated behavioral characteristics. Because the present framework targets neurophysiological EEG patterns rather than behavioral artifact signatures, such segments were treated as nuisance contamination. Nevertheless, future work should evaluate alternative retention thresholds and artifact-aware modeling strategies to determine whether clinically relevant information is inadvertently removed.

Several limitations need to be considered. The sample size of CAUEEG is modest: 1187/1379 recordings, by deep learning standards, which requires aggressive regularization. Transfer learning from large-scale EEG datasets may improve generalization. The use of a single-center dataset with a fixed 19-channel 10–20 montage also limits conclusions regarding cross-device and cross-site generalization. Differences in amplifier characteristics, electrode impedance, referencing schemes, sampling conditions, and available channel configurations may produce distribution shifts that are not fully removed by channel-wise normalization. Moreover, CEFA-Net was trained using a fixed 19-channel input, and its performance under reduced or alternative electrode montages was not directly evaluated. Future multicenter studies should therefore investigate acquisition harmonization, montage mapping or channel masking, calibration-based fine-tuning, and domain-adaptation strategies to improve robustness across EEG devices and clinical sites. The 590+ biomarkers were not exhaustively optimized; feature selection or emerging biomarkers such as network connectivity, microstates, and cross-frequency coupling may lead to better performance. Our study focused only on resting-state EEG; task-based ERPs provide complementary information. Full explainability is limited, advanced techniques, and clinicians facing visualization tools are required. This retrospective analysis needs prospective validation in real-world clinical workflows.

Future work should be directed at several promising directions: Multi-modal integration will integrate EEG with neuropsychological scores, genetic risk factors, MRI volumetrics, and blood biomarkers through cross-attention mechanisms 3. The continual learning framework will allow for incremental adaptation without catastrophic forgetting. The differential diagnosis capability of the framework will be able to distinguish between Alzheimer’s and other dementia subtypes. Longitudinal monitoring will track the disease trajectory to predict cognitive decline 56. Federated learning frameworks will collaborate on training across hospitals without sharing raw patient data. Uncertainty quantification methods will flag high-uncertainty cases for expert review. Finally, model compression techniques will reduce the computational requirements for point-of-care diagnosis in resource-limited settings.

## 5. Conclusions

This work investigated the automated detection of dementia from EEG signals and proposed a novel hybrid ensemble architecture, CEFA-Net, which synergistically integrates automatic deep learning-based feature extraction with explicit clinical domain knowledge. We conducted extensive experiments on the large-scale CAUEEG dataset, demonstrating that CEFA-Net achieves state-of-the-art performance: an accuracy of 81.02% for three-class dementia staging and 87.15% for binary abnormality detection, representing substantial gains over baseline methods by 6.75 to 9.10 percentage points. More importantly, our task-specific optimization strategies substantially improved the F1-score for mild cognitive impairment detection to as high as 78% (up 14 percentage points over baseline performance), thereby bridging an important gap in the literature and enhancing clinical utility for early intervention.

Our findings strongly suggest that hybrid approaches, combining learned and engineered representations, outperform pure data-driven and pure feature-based approaches in medical diagnostic tasks, which are typically characterized by small training datasets and well-established biomarkers. Ablation studies performed in a principled manner quantified standalone contributions of various architectural components, such as multi-branch CNN design, clinical feature incorporation, and ensemble learning, to name a few, and state-of-the-art training strategies, such as focal loss and class-aware augmentation, and provided practical insights for developers of similar medical AI systems. Finally, the clinical feasibility of CEFA-Net, given its inference times of 15–80 milliseconds per epoch with modest storage requirements, presents it as suitable for clinical deployment in screening and diagnostic workflows.

This work thus points to a number of promising future directions, including multi-modal data integration, differential diagnosis of dementia subtypes, monitoring of longitudinal progression, federated learning across institutions, and translation to point-of-care devices. Limitations persist regarding dataset size, single-center validation, and complete model interpretability; however, our results mark an important advance toward the goal of accurate, efficient, and accessible automated dementia detection. As the global burden of dementia continues to rise, computational methods such as CEFA-Net may prove to play an increasingly important role in facilitating early diagnosis, informing clinical decision-making, and improving outcomes for millions of patients worldwide.

The success of CEFA-Net speaks to a larger principle in medical AI: domain expertise and algorithmic sophistication are not paradigms in competition with one another but complementary partners. By thoughtfully integrating decades of neurophysiological research into state-of-the-art deep learning architectures, we can build diagnostic systems that are accurate, clinically aligned, interpretable by practitioners, and trustworthy. This collaboration between humans and AI—where machines augment and not replace expert judgment—represents the most promising path forward for realizing the full potential of artificial intelligence in healthcare.

## Figures and Tables

**Table 1:** Summary of CAUEEG dataset characteristics.

Characteristic	Details
Total Recordings	1379
Unique Patients	1155
Patient Age (years)	70.77 ± 9.90 (mean ± SD)
Gender Ratio (F:M)	100:60
Recording Duration (min)	13.34 ± 2.83 (mean ± SD)
EEG Channels	19 (10–20 system)
Sampling Frequency	200 Hz
Analog Filtering	0.5–70 Hz band-pass
Reference Scheme	Common average reference
Recording State	Awake, eyes closed, resting
Dementia Task Classes	Normal (459), MCI (417), Dementia (311)
Abnormal Task Classes	Normal (459), Abnormal (920)
Data Splits	Training (80%), Validation (10%), Test (10%)

**Table 2:** Performance of traditional machine learning methods.

Method	CAUEEG-Dementia (3-Class)			CAUEEG-Abnormal (2-Class)	
	Accuracy	Macro F1	Training Time	Accuracy	Macro F1
Gradient Boosting	0.6949	0.6888	32 min	0.7482	0.7398
SVM (RBF)	0.6780	0.6712	45 min	0.7321	0.7189
Logistic Regression	0.6441	0.6354	12 min	0.7117	0.6988
k-Nearest Neighbors	0.6610	0.6523	8 min	0.7234	0.7102
Best ML (Random Forest)	0.7259	0.7250	28 min	0.7805	0.7743

**Table 3:** Class-specific performance of best ML method (Random Forest).

Task	Class	Precision	Recall	F1-Score	Support
Dementia	Normal	0.77	0.82	0.80	45
	MCI	0.65	0.64	0.64	39
Abnormal	Dementia	0.76	0.70	0.73	34
	Normal	0.72	0.59	0.65	45
	Abnormal	0.80	0.88	0.84	91

**Table 4:** Performance of 1D-CNN architectures.

Architecture	CAUEEG-Dementia (3-Class)		CAUEEG-Abnormal (2-Class)	
	Accuracy	Macro F1	Accuracy	Macro F1
Standard CNN	0.7124	0.7072	0.8230	0.8245
Deep CNN	0.6966	0.6895	0.8017	0.7987
ResNet CNN	0.7427	0.7416	0.8201	0.8186
Attention CNN	0.7225	0.7214	0.8085	0.8002
Best 1D-CNN	0.7427	0.7416	0.8230	0.8245

**Table 5:** Performance of CEFA-Net (proposed method).

Model	Task	Accuracy	Macro F1	95% CI	Training Time
CEFA-Net-B	Abnormal (2-class)	0.8715	0.8741	[0.862–0.881]	8.5 h
CEFA-Net-M	Dementia (3-class)	0.8102	0.8115	[0.798–0.823]	9.2 h

**Table 6:** Class-specific performance of CEFA-Net models.

Model	Class	Precision	Recall	F1-Score
CEFA-Net-B (Abnormal)	Normal	0.84	0.83	0.84
	Abnormal	0.89	0.85	0.87
CEFA-Net-M (Dementia)	Normal	0.86	0.82	0.84
	MCI	0.77	0.79	0.78
	Dementia	0.82	0.85	0.84

**Table 7:** Patient-disjoint performance on no-overlap splits.

Task	Evaluation Setting	Accuracy	Macro F1	Max Segments	TTA Augmentations
Abnormal detection	Aggregated level	0.8540	0.8555	10	5
Dementia staging	Aggregated level	0.7380	0.7395	10	5

**Table 8:** Performance of individual CEFA-Net components.

Component	Dementia Task		Abnormal Task	
	Accuracy	Macro F1	Accuracy	Macro F1
Hybrid CNN + Clinical	0.7797	0.7802	0.8520	0.8531
ResNet CNN + Clinical	0.7881	0.7893	0.8612	0.8629
Attention CNN + Clinical	0.7966	0.7978	0.8583	0.8597
Full Ensemble	0.8102	0.8115	0.8715	0.8741
Ensemble Gain	+1.36%	+1.37%	+1.03%	+1.12%

**Table 9:** Ablation study results for CEFA-Net-M (dementia task).

Configuration	Accuracy	Macro F1	MCI F1	Δ vs. Full
Full CEFA-Net-M	0.8102	0.8115	0.78	baseline
Clinical Features	0.7542	0.7529	0.70	−5.60%
Ensemble (single best)	0.7966	0.7978	0.76	−1.36%
Focal Loss (CE loss)	0.7729	0.7712	0.71	−3.73%
Class-aware Augmentation	0.7814	0.7801	0.73	−2.88%
Test-Time Augmentation	0.7966	0.7953	0.76	−1.36%
Only Clinical Features (no CNN)	0.7259	0.7250	0.64	−8.43%
Only CNN (no Clinical)	0.7427	0.7416	0.69	−6.75%

**Table 10:** Comparison with other studies on CAUEEG dataset.

Method	Year	Approach	Dementia Acc. (3-Class)
Kim et al. (CEEDNet) [16]	2023	Deep CNN (ResNet-18 + STFT)	74.66%
Barbera et al. [17]	2024	Lightweight GNN (22K params)	61.86%
Zhang et al. (Bi-MCGNN) [18]	2025	Dual-Path GNN (spatial-temporal + spatial-spectral)	80.21% ± 0.76
CEFA-Net-M (Proposed)	2025	Hybrid CNN-Clinical Ensemble	81.02% ± 1.23

## Data Availability

The data analyzed in this study are from the Chung-Ang University Hospital EEG dataset. The CAUEEG dataset is available for academic and research purposes upon request through the official dataset repository, subject to approval requirements specified by the data providers https://github.com/ipis-mjkim/caueeg-dataset (accessed on 12 November 2025).

## Abbreviations

The following abbreviations are used in this manuscript:

AD	Alzheimer’s disease
AI	Artificial intelligence
CAUEEG	Chung-Ang University Hospital EEG dataset
CEFA-Net	Clinical EEG Feature-Augmented Network
CNN	Convolutional neural network
DAR	Delta/alpha ratio
EEG	Electroencephalography
FIR	Finite impulse response
F1	F1-score
GAP	Global average pooling
GB	Gradient Boosting
kNN	k-Nearest Neighbors
LR	Logistic Regression
MCI	Mild cognitive impairment
ML	Machine learning
PSD	Power spectral density
RF	Random Forest
SE	Squeeze-and-Excitation
SNR	Signal-to-noise ratio
SOTA	State of the art
SVM	Support Vector Machine
TAR	Theta/alpha ratio
TTA	Test-time augmentation

## References

[1] Martí-Navia A., Álvarez-Sánchez L., Ferré-González L., López A., Peña-Bautista C., Balaguer Á., Pedrosa N., Vico H., Baquero M., Cháfer-Pericás C. (2025). Clinical Validation of New Alzheimer Disease Diagnosis Tools Based on Plasma P-Tau217. Sci. Rep..

[2] Mattsson-Carlgren N., Collij L.E., Stomrud E., Pichet Binette A., Ossenkoppele R., Smith R., Karlsson L., Lantero-Rodriguez J., Snellman A., Strandberg O. (2024). Plasma Biomarker Strategy for Selecting Patients with Alzheimer Disease for Antiamyloid Immunotherapies. JAMA Neurol..

[3] El Abiad E., Al-Kuwari A., Al-Aani U., Al Jaidah Y., Chaari A. (2024). Navigating the Alzheimer’s Biomarker Landscape: A Comprehensive Analysis of Fluid-Based Diagnostics. Cells.

[4] Ranjan S., Jain A., Badal R., Kumar A., Shende H., Joshi D., Yadav P., Kumar L. (2025). Working Memory Functional Connectivity Analysis for Dementia Classification Using EEG. arXiv.

[5] Babiloni C., Arakaki X., Azami H., Bennys K., Blinowska K., Bonanni L., Bujan A., Carrillo M.C., Cichocki A., de Frutos-Lucas J. (2021). Measures of Resting State EEG Rhythms for Clinical Trials in Alzheimer’s Disease: Recommendations of an Expert Panel. Alzheimers Dement..

[6] Musaeus C.S., Engedal K., Høgh P., Jelic V., Mørup M., Naik M., Oeksengaard A.R., Snaedal J., Wahlund L.O., Waldemar G. (2019). Oscillatory Connectivity as a Diagnostic Marker of Dementia Due to Alzheimer’s Disease. Clin. Neurophysiol..

[7] Brown C.W., Chen H.Y., Panegyres P.K. (2023). Electroencephalography in Young Onset Dementia. BMC Neurol..

[8] Zheng X., Wang B., Liu H., Wu W., Sun J., Fang W., Jiang R., Hu Y., Jin C., Wei X. (2023). Diagnosis of Alzheimer’s Disease via Resting-State EEG: Integration of Spectrum, Complexity, and Synchronization Signal Features. Front. Aging Neurosci..

[9] Roy Y., Banville H., Albuquerque I., Gramfort A., Falk T.H., Faubert J. (2019). Deep Learning-Based Electroencephalography Analysis: A Systematic Review. J. Neural Eng..

[10] Latif S., Islam N.U., Uddin Z., Cheema K.M., Ahmed S.S., Khan M.F. (2025). Deep Ensemble Learning with Transformer Models for Enhanced Alzheimer’s Disease Detection. Sci. Rep..

[11] Raj V.A., Parupudi T., Thalengala A., Nayak S.G. (2025). A Comprehensive Review of Deep Learning Models for Denoising EEG Signals: Challenges, Advances, and Future Directions. Discov. Appl. Sci..

[12] Ding J.-E., Zilverstand A., Yang S., Yang A.C.-C., Liu F. (2025). Variational Mixture of Graph Neural Experts for Alzheimer’s Disease Biomarker Recognition in EEG Brain Networks. arXiv.

[13] Akbar F., Alkhrijah Y., Usman S.M., Khalid S., Ihsan I., Alawad M.A. (2025). NeuroFusionNet: A Hybrid EEG Feature Fusion Framework for Accurate and Explainable Alzheimer’s Disease Detection. Sci. Rep..

[14] Alayba A.M., Senan E.M., Alshudukhi J.S. (2024). Enhancing Early Detection of Alzheimer’s Disease through Hybrid Models Based on Feature Fusion of Multi-CNN and Handcrafted Features. Sci. Rep..

[15] Zhang J., Zheng S., Chen W., Du G., Fu Q., Jiang H. (2024). A Scheme Combining Feature Fusion and Hybrid Deep Learning Models for Epileptic Seizure Detection and Prediction. Sci. Rep..

[16] Kim M.J., Youn Y.C., Paik J. (2023). Deep Learning-Based EEG Analysis to Classify Normal, Mild Cognitive Impairment, and Dementia: Algorithms and Dataset. Neuroimage.

[17] Barbera T., Zini S., Bianco S., Napoletano P. Lightweight Graph Neural Network for Dementia Assessment from EEG Recordings. Proceedings of the 8th International Forum on Research and Technologies for Society and Industry Leveraging a Better Tomorrow 2024.

[18] Zhang D., Zhu C. (2025). A Dual Path Graph Neural Network Framework for Dementia Diagnosis. Sci. Rep..

[19] Hata M., Yanagisawa T., Miyazaki Y., Omori H., Hirashima A., Nakagawa Y., Eto M., Yoshiyama K., Kanemoto H., Nyamradnaa B. (2025). Accurate Deep-Learning Model to Differentiate Dementia Severity and Diagnosis Using a Portable Electroencephalography Device. Sci. Rep..

[20] Akbar F., Alkhrijah Y., Usman S.M., Khalid S., Ihsan I., Alawad M.A. (2026). A Deep-SVM Hybrid Framework with Enhanced EEG Feature Engineering and SHAP-Based Explainability for Alzheimer’s Classification. Sci. Rep..

[21] He K., Zhang X., Ren S., Sun J. Deep Residual Learning for Image Recognition. Proceedings of the IEEE Computer Society Conference on Computer Vision and Pattern Recognition.

[22] Hu J., Shen L., Sun G. Squeeze-and-Excitation Networks. Proceedings of the IEEE Computer Society Conference on Computer Vision and Pattern Recognition.

[23] Özçelik K.N.Ç., Özçelik S.T.A., Timurkaan S. (2026). Machine Learning-Based Risk Prediction for Feline Mammary Tumours: A Comprehensive Epidemiological Analysis Using Multi-Model Ensemble Approach. Vet. Comp. Oncol..

[24] Al-Qazzaz N.K., Ali S.H.B.M., Ahmad S.A., Islam M.S., Escudero J. (2018). Discrimination of Stroke-Related Mild Cognitive Impairment and Vascular Dementia Using EEG Signal Analysis. Med. Biol. Eng. Comput..

[25] Klem G., Lüders H., Jasper H., Elger C. (1999). The Ten-Twenty Electrode System of the International Federation. Int. Fed. Clin. Neurophysiol. Electroencephalogr. Clin. Neurophysiol. Suppl..

[26] McKhann G., Drachman D., Folstein M., Katzman R., Price D., Stadlan E.M. (1984). Clinical Diagnosis of Alzheimer’s Disease: Report of the NINCDS-ADRDA Work Group under the Auspices of Department of Health and Human Services Task Force on Alzheimer’s Disease. Neurology.

[27] Petersen R.C. (2004). Mild Cognitive Impairment as a Diagnostic Entity. J. Intern. Med..

[28] Varoquaux G., Raamana P.R., Engemann D.A., Hoyos-Idrobo A., Schwartz Y., Thirion B. (2017). Assessing and Tuning Brain Decoders: Cross-Validation, Caveats, and Guidelines. Neuroimage.

[29] Jack C.R., Knopman D.S., Jagust W.J., Petersen R.C., Weiner M.W., Aisen P.S., Shaw L.M., Vemuri P., Wiste H.J., Weigand S.D. (2013). Tracking Pathophysiological Processes in Alzheimer’s Disease: An Updated Hypothetical Model of Dynamic Biomarkers. Lancet Neurol..

[30] Hu L., Zhang Z. (2019). EEG Signal Processing and Feature Extraction.

[31] Widmann A., Schröger E., Maess B. (2015). Digital Filter Design for Electrophysiological Data—A Practical Approach. J. Neurosci. Methods.

[32] Babiloni C., Blinowska K., Bonanni L., Cichocki A., De Haan W., Del Percio C., Dubois B., Escudero J., Fernández A., Frisoni G. (2020). What Electrophysiology Tells Us about Alzheimer’s Disease: A Window into the Synchronization and Connectivity of Brain Neurons. Neurobiol. Aging.

[33] Yao D. (2001). A Method to Standardize a Reference of Scalp EEG Recordings to a Point at Infinity. Physiol. Meas..

[34] Cohen M.X. (2014). Analyzing Neural Time Series Data: Theory and Practice. Analyzing Neural Time Series Data: Theory and Practice.

[35] Ioffe S., Szegedy C. Batch Normalization: Accelerating Deep Network Training by Reducing Internal Covariate Shift. Proceedings of the 32nd International Conference on Machine Learning, ICML 2015.

[36] Jeong J. (2004). EEG Dynamics in Patients with Alzheimer’s Disease. Clin. Neurophysiol..

[37] Huang C., Wahlund L.O., Dierks T., Julin P., Winblad B., Jelic V. (2000). Discrimination of Alzheimer’s Disease and Mild Cognitive Impairment by Equivalent EEG Sources: A Cross-Sectional and Longitudinal Study. Clin. Neurophysiol..

[38] Klimesch W. (1999). EEG Alpha and Theta Oscillations Reflect Cognitive and Memory Performance: A Review and Analysis. Brain Res. Rev..

[39] Garn H., Coronel C., Waser M., Caravias G., Ransmayr G. (2017). Differential Diagnosis between Patients with Probable Alzheimer’s Disease, Parkinson’s Disease Dementia, or Dementia with Lewy Bodies and Frontotemporal Dementia, Behavioral Variant, Using Quantitative Electroencephalographic Features. J. Neural Transm..

[40] Prichep L.S., John E.R., Ferris S.H., Reisberg B., Almas M., Alper K., Cancro R. (1994). Quantitative EEG Correlates of Cognitive Deterioration in the Elderly. Neurobiol. Aging.

[41] Cassani R., Estarellas M., San-Martin R., Fraga F.J., Falk T.H. (2018). Systematic Review on Resting-State EEG for Alzheimer’s Disease Diagnosis and Progression Assessment. Dis. Markers.

[42] Babiloni C., Binetti G., Cassetta E., Forno G.D., Del Percio C., Ferreri F., Ferri R., Frisoni G., Hirata K., Lanuzza B. (2006). Sources of Cortical Rhythms Change as a Function of Cognitive Impairment in Pathological Aging: A Multicenter Study. Clin. Neurophysiol..

[43] Sharma M., Dhere A., Pachori R.B., Gadre V.M. (2017). An Automatic Detection of Focal EEG Signals Using New Class of Time–Frequency Localized Orthogonal Wavelet Filter Banks. Knowl. Based. Syst..

[44] Ruiz-Gómez S.J., Gómez C., Poza J., Gutiérrez-Tobal G.C., Tola-Arribas M.A., Cano M., Hornero R. (2018). Automated Multiclass Classification of Spontaneous EEG Activity in Alzheimer’s Disease and Mild Cognitive Impairment. Entropy.

[45] Uyanık H., Ozcelik S.T.A., Duranay Z.B., Sengur A., Acharya U.R. (2022). Use of Differential Entropy for Automated Emotion Recognition in a Virtual Reality Environment with EEG Signals. Diagnostics.

[46] Breiman L. (2001). Random Forests. Mach. Learn..

[47] Friedman J.H. (2001). Greedy Function Approximation: A Gradient Boosting Machine. Ann. Stat..

[48] Cortes C., Vapnik V. (1995). Support-Vector Networks. Mach. Learn..

[49] Cox D.R. (1958). The Regression Analysis of Binary Sequences. J. R. Stat. Soc. Ser. B Stat. Methodol..

[50] Cover T.M., Hart P.E. (1967). Nearest Neighbor Pattern Classification. IEEE Trans. Inf. Theory.

[51] Lin M., Chen Q., Yan S. Network In Network. Proceedings of the 2nd International Conference on Learning Representations, ICLR 2014, Conference Track Proceedings.

[52] Woo S., Park J., Lee J.-Y., Kweon I.S. (2018). CBAM: Convolutional Block Attention Module. CBAM: Convolutional Block Attention Module, Proceedings of the 15th European Conference, Munich, Germany, 8–14 September 2018.

[53] Kingma D.P., Ba J.L. Adam: A Method for Stochastic Optimization. Proceedings of the 3rd International Conference on Learning Representations, ICLR 2015—Conference Track Proceedings.

[54] McNemar Q. (1947). Note on the Sampling Error of the Difference between Correlated Proportions or Percentages. Psychometrika.

[55] Wen T., Zhang Z. (2018). Deep Convolution Neural Network and Autoencoders-Based Unsupervised Feature Learning of EEG Signals. IEEE Access.

[56] Thodoroff P., Pineau J., Lim A. Learning Robust Features Using Deep Learning for Automatic Seizure Detection. Proceedings of the 1st Machine Learning for Healthcare Conference, PMLR.

[57] Supratak A., Dong H., Wu C., Guo Y. (2017). DeepSleepNet: A Model for Automatic Sleep Stage Scoring Based on Raw Single-Channel EEG. IEEE Trans. Neural Syst. Rehabil. Eng..

[58] Croatti A., Gabellini M., Montagna S., Ricci A. (2020). On the Integration of Agents and Digital Twins in Healthcare. J. Med. Syst..

[59] Ravi D., Wong C., Deligianni F., Berthelot M., Andreu-Perez J., Lo B., Yang G.Z. (2017). Deep Learning for Health Informatics. IEEE J. Biomed. Health Inform..

[60] Duboisa B., Padovanib A., Scheltensc P., Rossid A., Agnello G.D. (2016). Timely Diagnosis for Alzheimer’s Disease: A Literature Review on Benefits and Challenges. J. Alzheimers Dis..

